# Molecular decay of enamel matrix protein genes in turtles and other edentulous amniotes

**DOI:** 10.1186/1471-2148-13-20

**Published:** 2013-01-23

**Authors:** Robert W Meredith, John Gatesy, Mark S Springer

**Affiliations:** 1 Department of Biology, University of California, Riverside, CA 92521, USA; 2 Current address: Department of Biology and Molecular Biology, Montclair State University, Montclair, NJ, 07043, USA

**Keywords:** Ameloblastin, Amelogenin, Enamel matrix protein genes, Enamelin, Pseudogenes, Testudines

## Abstract

**Background:**

Secondary edentulism (toothlessness) has evolved on multiple occasions in amniotes including several mammalian lineages (pangolins, anteaters, baleen whales), birds, and turtles. All edentulous amniote clades have evolved from ancestors with enamel-capped teeth. Previous studies have documented the molecular decay of tooth-specific genes in edentulous mammals, all of which lost their teeth in the Cenozoic, and birds, which lost their teeth in the Cretaceous. By contrast with mammals and birds, tooth loss in turtles occurred in the Jurassic (201.6-145.5 Ma), providing an extended time window for tooth gene degradation in this clade. The release of the painted turtle and Chinese softshell turtle genomes provides an opportunity to recover the decayed remains of tooth-specific genes in Testudines.

**Results:**

We queried available genomes of Testudines (*Chrysemys picta* [painted turtle], *Pelodiscus sinensis* [Chinese softshell turtle]), Aves (*Anas platyrhynchos* [duck], *Gallus gallus* [chicken], *Meleagris gallopavo* [turkey], *Melopsittacus undulatus* [budgerigar], *Taeniopygia guttata* [zebra finch]), and enamelless mammals (*Orycteropus afer* [aardvark], *Choloepus hoffmanni* [Hoffmann’s two-toed sloth], *Dasypus novemcinctus* [nine-banded armadillo]) for remnants of three enamel matrix protein (EMP) genes with putative enamel-specific functions. Remnants of the *AMBN* and *ENAM* genes were recovered in *Chrysemys* and retain their original synteny. Remnants of *AMEL* were recovered in both testudines, although there are no shared frameshifts. We also show that there are inactivated copies of *AMBN*, *AMEL* and *ENAM* in representatives of divergent avian lineages including Galloanserae, Passeriformes, and Psittaciformes, and that there are shared frameshift mutations in all three genes that predate the basal split in Neognathae. Among enamelless mammals, all three EMP genes exhibit inactivating mutations in *Orycteropus* and *Choloepus*.

**Conclusions:**

Our results highlight the power of combining fossil and genomic evidence to decipher macroevolutionary transitions and characterize the functional range of different loci involved in tooth development. The fossil record and phylogenetics combine to predict the occurrence of molecular fossils of tooth-specific genes in the genomes of edentulous amniotes, and in every case these molecular fossils have been discovered. The widespread occurrence of EMP pseudogenes in turtles, birds, and edentulous/enamelless mammals also provides compelling evidence that in amniotes, the only unique, non-redundant function of these genes is in enamel formation.

## Background

Gnathostomes are unique among vertebrates in utilizing jaws and teeth for food acquisition and processing. Divergent gnathostome lineages exhibit a wide array of dental modifications in association with diverse dietary specializations. Multiple iterations of secondary tooth loss have also evolved in gnathostomes, perhaps most famously in birds, turtles, and several mammalian lineages including baleen whales, pangolins, and anteaters [1-3]. There are also mammalian species with enamelless teeth, i.e., sloths, armadillos, aardvarks, pygmy and dwarf sperm whales [2].

Tooth development is an intricate process that encompasses a complex series of epithelial-mesenchymal interactions involving growth factors, transcription factors, and signal receptors that affect tooth shape, tooth number, and cusp number [4-9]. Several hundred genes are associated with tooth development [6], including members of the hedgehog (Hh), fibroblast growth factor (Fgf) and bone morphogenic protein (Bmp) families that mediate epithelio-mesenchymal signaling interactions [10]. Most or all of the genes that are involved in early tooth development are thought to be pleiotropic and have additional functions outside of tooth formation. However, there are also genes with putative tooth-specific functions that are expressed later in development by dentin-forming odontoblasts and/or enamel-forming ameloblasts [11-17]. These genes affect the physical properties of teeth including enamel thickness and structure. The durability of teeth, in combination with tooth-specific genes that impact the physical structure of dentin and enamel, make teeth a model system for studying the coevolution of morphological change in the fossil record and molecular change in the genome.

Previous studies have shown that the genes encoding three enamel matrix proteins (EMPs), enamelin, amelogenin, and ameloblastin, have become pseudogenized in one or more edentulous/enamelless mammals by frameshift mutations and/or stop codons [1-3] (Table 1). Further, the molecular decay of tooth-specific genes in placental mammal lineages has been shown to parallel the morphological degeneration of enamel in the fossil record [1-3]. The retention of inactivated EMP genes in mammalian lineages is not surprising given that all edentulous and enamelless taxa appear to have originated in the Cenozoic [2].

**Table 1:** Pseudogene remnants of enamel matrix protein (EMP) genes in edentulous and enamelless amniotes

**Tooth gene**	**Taxon**		
	**Turtles**	**Birds**	**Mammals**
1. *ENAM*	Frameshift mutations in *Chrysemys *exon 9 (Additional file 1)	Frameshift mutations in exon 9 of *Anas*, *Gallus*, *Meleagris*, *Melopsittacus*, and *Taeniopygia* (Additional file 1; also see Al-Hashimi et al. [18])	Frameshift mutations in exon 10 (homologous to exon 9 of *Alligator*) of Tubulidentata (aardvark), Pholidota (pangolins), Xenarthra (anteaters, sloths, armadillos), Mysticeti (baleen whales), and Kogiidae (pygmy and dwarf sperm whales) [1,2]
2. *AMBN*	Frameshift mutations in *Chrysemys picta *exons 6 and 11 (Additional file 3)	Frameshift mutations in *Anas platyrhynchos *(exons 6, 8), *Gallus gallus *(exons 3, 6), and *Taeniopygia guttata* (exon 6); stop codon in exon 8 of *Melopsittacus undulatus* (Additional file 3)	Frameshift mutations in exon 13 of seven baleen whales (Mysticeti) [1,3]; premature stop codon in exon 13 of *Monodon monoceros*[19]; multiple inactivating mutations in *Orycteropus afer* (stop codon in exon 5, complete deletion of exon 6, frameshift deletion in exon 13) (Additional file 5); acceptor splice site mutation in intron 2 of *Choloepus hoffmanni *(AG to AT)
3. *AMEL*	Framshift mutations in *Chrysemys picta *(exon 1) and *Pelodiscus sinensis *(exons 1, 3, 4) (Additional file 7)	Frameshift mutations in *Anas platyrhynchos *(exons 1, 2, 3, 4), *Gallus gallus *(exons 1, 2, 4), *Meleagris gallopavo* (exons 1, 2), *Melopsittacus undulatus *(exons 1, 2), and *Taeniopygia guttata* (exons 2) (Additional file 7; also see Sire et al. [20] and Davit-Béal et al. [21])	Frameshift mutations in *Choloepus hoffmanni *(exons 2, 6), deletion of 3’ end of intron 6 (including splice site) and first 14 bp of exon 7 in *Dasypus novemcinctus*, and two stop codons in exon 6 of *Orycteropus afer *(Additional file 11); frameshift mutations in exon 6 of five mysticetes [1,3]

By contrast, tooth loss in both birds and turtles occurred in the Mesozoic, providing a longer time period for the molecular decay of tooth-specific genes in these lineages. In birds, the presence of a horny beak and gizzard have presumably compensated for edentulism in food acquisition and processing [22]. Edentulism in the ancestry of modern birds occurred in the Cretaceous, between 125 and 65.5 Ma, and also evolved independently in several lineages of extinct birds that are on the stem to Neornithes (crown group birds) [22]. Even though tooth loss in Neornithes occurred in the Cretaceous, the chicken (*Gallus gallus*) genome retains pseudogenized copies of both *AMEL* and *ENAM*[18,20,21].

Edentulism in Testudines (turtles) occurred even earlier, at least as far back as the Late Jurassic [23,24]. The oldest turtle is *Odontochelys semitestacea* from the Triassic of China (~220 Ma) [25]. Marginal and palatal teeth are both present in this taxon [25]. Slightly younger (~210 Ma) is *Proganochelys quenstedti* from the Upper Triassic [23,26]. Marginal teeth are absent in *Proganochelys*, but palatal teeth are present. Phylogenetic analyses suggest that *Proganochelys*, like *Odontochelys*, is a stem testudine [23]. The oldest testudines with unambiguous crown-group affinities, including tooth loss on the palatines, vomer, and pterygoids, are from the Late Jurassic [23].

To date, remnants of pseudogenized copies of tooth-specific genes in Testudines have not been reported. Girondot and Sire [27] were unable to amplify fragments of amelogenin in turtles with degenerate PCR primers, and it remains unclear if vestiges of tooth-specific genes are retained in testudine genomes given the antiquity of tooth loss in this clade. On the other hand, phylogenomic data suggest that Testudines have slower rates of nuclear gene evolution than birds and mammals (fig. 1 in [28]). The recent release of two testudine genomes, *Chrysemys picta* (painted turtle) and *Pelodiscus sinensis* (Chinese softshell turtle), provides an opportunity to screen for remnants of tooth-specific genes in turtles. These taxa, both of which belong to Cryptodira (hidden neck turtles), index the basal cladogenic event among crown cryptodires and leave only Pleurodira (sideneck turtles) unrepresented among the three oldest crown-testudine lineages. Cryptodira and Pleurodira have traditionally been regarded as reciprocally monophyletic based on morphology [29-31] and large molecular data sets [32,33]. An exception is Barley et al.’s [32] coalescence analysis, which recovered a basal split between softshells plus the pig-nosed turtle (*Carettochelys insculpta*) and Pleurodira plus other Cryptodira. However, Barley et al.’s [32] concatenation analysis recovered Cryptodira and Pleurodira. Moreover, the most complete testudine phylogeny with a variety of outgroups [33] supports the monophyly of both Cryptodira and Pleurodira.

Here, we report the results of querying the genomes of *Chrysemys* and *Pelodiscus* for inactivated remnants of three EMP genes, *ENAM*, *AMEL*, and *AMBN*, all of which are hypothesized to have tooth-specific functions. We also report the results of querying genome sequences of five birds (*Gallus gallus* [chicken], *Meleagris gallopavo* [turkey], *Taeniopygia guttata* [zebra finch], *Anas platyrhynchos* [duck], *Melopsittacus undulatus* [budgerigar]) and three enamelless mammals (*Orycteropus afer* [aardvark], *Choloepus hoffmanni* [Hoffmann’s two-toed sloth], *Dasypus novemcinctus* [nine-banded armadillo]) for remnants of these EMP genes. The occurrence of pseudogenized remnants of EMP genes in highly divergent amniotes, including turtles, birds, and edentulous/enamelless mammals, would provide robust evidence for the hypothesis that the only essential, non-redundant function of *AMEL*, *AMBN*, and *ENAM* is in enamel formation. Moreover, the search for shared frameshift mutations in EMP genes provides an opportunity to date the timing of enamel loss in these clades.

## Methods

The genomes of *Chrysemys picta* (painted turtle; PreEnsembl) and *Pelodiscus sinensis* (Chinese softshell turtle; Ensembl 68) were queried with BLASTN using crocodylian mRNA sequences for three enamel matrix proteins genes: *Paleosuchus palpebrosus* (Cuvier’s dwarf caiman) *AMEL* (AF095568), *Caiman crocodilus* (spectacled caiman) *AMBN* (AY043290), and *Crocodylus niloticus* (Nile crocodile) *ENAM* (GU344683). Intron-exon boundaries of mRNA sequences were determined by blasting crocodylian mRNAs against the recently released genome sequence of *Alligator mississippiensis* (American alligator). Crocodylian sequences were chosen as probes based on recent evidence that provides strong support for a sister group relationship between Testudines and Archosauria (crocodylians, birds) [27,34]. *AMBN* has 11 exons in *Alligator*, all of which include protein-coding regions. *AMEL* includes six exons in *Alligator*, five of which (1–5) contain protein-coding regions. Finally, *ENAM* includes nine exons in *Alligator*, eight of which (2–9) include protein-coding regions. Remnants of EMP genes that were recovered from *Chrysemys* were subsequently used to query the *Pelodiscus* genome, and remnants of EMP genes from *Pelodiscus* were used to query the *Chrysemys* genome. Five bird genomes (*Anas platyrhynchos* [duck], *Gallus gallus* [chicken], *Meleagris gallopavo* [turkey], *Melopsittacus undulatus* [budgerigar], *Taeniopygia guttata* [zebra finch]) were queried with crocodylian mRNAs and virtual pseudogene mRNAs for *AMEL* (EU340348) and *ENAM* (GU198360) from *Gallus*, as well as with positive hits from the initial round of screening for the bird genomes. The NCBI genomes of three enamelless mammals (*Orycteropus afer* [aardvark], *Choloepus hoffmanni* [Hoffmann’s two-toed sloth], *Dasypus novemcinctus* [nine-banded armadillo]) were queried with gene sequences from mammals that retain enamel-capped teeth (i.e*., Bos taurus*, *Homo sapiens*, *Echinops telfairi*, *Elephantulus edwardii*, *Trichechus manatus*). Sequences were aligned with Se-Al [35].

**Figure 1:**
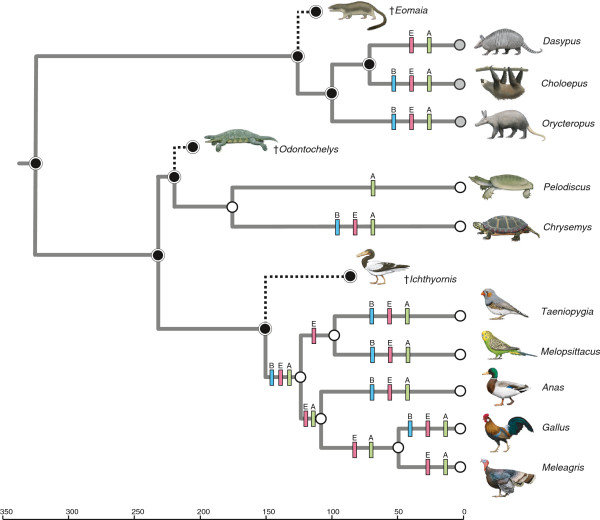
**Timetree of edentulous and enamelless amniote taxa for which genomic sequences are available (Ensembl, PreEnsembl, NCBI) with a mapping of inferred inactivating mutations (frameshifts, premature stop codons, splice site mutations) in three EMP genes to particular branches in the tree. **Branches with inactivating mutations are marked by vertical bars (pink = *ENAM *[E], blue = *AMBN *[B], green = *AMEL* [A]). Extinct stem taxa with teeth (*Odontochelys*, *Ichthyornis*, *Eomaia*) are also shown. Mutations were mapped to branches by Fitch parsimony with delayed transformation optimization. Teeth with enamel, teeth without enamel, and edentulism are denoted with black, gray, and white-filled circles, respectively, at terminal and internal nodes. In combination with fossil evidence, dN/dS ratios and frameshift mutations in *ENAM *suggest that enamel was lost independently in the common ancestor of Pilosa (sloths and anteaters) and in multiple armadillo lineages, including *Dasypus*[2]. Remnants of *AMBN *and *ENAM *were not recovered from the *Pelodiscus *genome. References for divergence dates and fossil ages are provided in Methods.

Selection analyses on branches were performed with PAML 4.5 [36] to estimate dN/dS values for an assemblage of 20 complete or nearly complete mammalian *AMBN* sequences, all of which were derived from Ensembl 68 (*Ailuropoda melanoleuca, Bos taurus, Callithrix jacchus, Equus caballus, Oryctolagus cuniculus*) or NCBI (*Canis lupus familiaris* [XM_539304], *Choloepus hoffmanni* [ABVD01066982, TI 1336370099, ABVD01066984, TI 1338793682, TI 1368206053, ABVD01066985, ABVD01066986], *Dasypus novemcinctus* [AAGV03237582], *Echinops telfairi* [AAIY02097297, AAIY02097298], *Elephantulus edwardii* [AMGZ01205754, AMGZ01205755], *Homo sapiens* [NM_016519], *Loxodonta africana* [AAGU03054364, AAGU03054363], *Orycteropus afer* [ALYB01124783], *Pan paniscus* [XM_001160377], *Pan troglodytes* [XM_001160377], *Pongo abelii* [XM_002814835], *Procavia capensis* [ABRQ01419216, ABRQ01419217, ABRQ01419218, ABRQ01419219, ABRQ01419220], *Sus scrofa* [NM_214037]). Sequences were aligned with Se-Al [35]. Branch analyses were performed with four branch categories: *Orycteropus*, *Choloepus*, *Dasypus*, and all other branches. *Orycteropus*, *Choloepus* and *Dasypus* were recognized as separate branches in the PAML runs based on the hypothesized independent loss of enamel in these lineages [2]. PAML analyses were conducted with codon frequency models 1, 2, and 3. We used a composite species tree based on Meredith et al. [37] and Springer et al. [38]. Approximate divergence dates and fossil ages in Figure 1 are taken from the following sources: Amniota and Testudines to Archosauria [39]; *Pelodiscus* to *Chrysemys*[40]; *Odontochelys*[25]; Neognathae [41]; Galloanserae and *Gallus* to *Meleagris*[42]; *Taeniopygia* to *Melopsittacus*[43]; *Ichthyornis*[44]; Xenarthra [45]; Xenarthra to *Orycteropus*[37]; *Eomaia*[46].

## Results

### ENAM Gene

Blast searches with *Crocodylus niloticus* (Nile crocodile) *ENAM* (GU344683) identified almost the entirety of exon 9 in *Chrysemys picta* (Additional file 1). Remnants of this exon in *C. picta* were identified on scaffold JH584398, which has not yet been mapped onto a chromosome. The *ENAM* sequence for exon 9 in *Chrysemys* includes numerous frameshift indels (Figure 1, Table 2, Additional files 1, 2), one of which is a SINE insertion that shares homology with members of the Cry family of SINEs in Cryptodira [47] (Additional files 1, 2). An additional SINE insertion occurs 3’ to the stop codon. Remnants of exon 9 in *Chrysemys* comprise ~3840 bp after excluding the SINE insertions. *Chrysemys* exon 9 retains a “TAA” stop codon in the same location as *Crocodylus*. By contrast with the results of blast searches against the *Chrysemys* genome, blast searches with *Crocodylus ENAM* resulted in no significant hits to the *Pelodiscus sinensis* genome. Likewise, blast searches with the recovered *ENAM* pseudogene segment from *Chrysemys* did not find significant matches in the *Pelodiscus* genome.

**Table 2:** Examples of inactivating mutations in enamel matrix protein genes among edentulous and enamelless amniotes with genome sequences

**Taxon**	**Inactivating mutations**		
	***ENAM***	***AMBN***	***AMEL***
Cryptodira	None	None	Possible shared frameshift mutations in exon 2 (see AF9)
*Chrysemys*	AF1 Insertions: 65, 271, 411, 485, 994-1138 (Cry), 2526-2529, 2559-2571, 3268	AF3 Insertions: 956-957, 972, 1051-1052	AF7 Deletion: 106
	AF1 Deletions: 588, 1264-1276, 3325-3343	AF3 Deletions: 497-498, 605-611, 794-804, 984-985	
*Pelodiscus*	Gene not recovered	Gene not recovered	AF7 Deletions: 122-125, 208, 271, 276
Neognathae	AF1 Insertions: 1985-1989, 2143-2144,	AF3 Deletion: 515-524	AF7 Deletions: 134 (with subsequent deletion of one additional bp in *Taeniopygia*), 242, 709-710
	AF1 Deletions: 2634-2637, 3353-3758		
Galloanserae	AF1 Deletion: 1189-1192	None	AF7 Deletions: 173-174, 282-289
*Anas*	AF1 Insertions: 552-562, 2031-2040	AF3 Deletions: 216-217, 226, 455, 727-728	AF7 Insertions: 152, 181
	AF1 Deletions: 2626-2627		AF7 Deletion: 88-94
*Gallus* + *Meleagris*	AF1 Insertion: 2654	None	AF7 Insertion: 484
	AF1 Deletion: 3263-3267		
*Gallus*	AF1 Insertions: 512, 930-931	AF3 Insertions: 225, 230	AF7 Deletion: 275-276
	AF1 Deletions: 126-127, 585-595		
*Meleagris*	AF1 Insertion: 2438-2441	Gene not recovered	AF7 Deletion: 359
*Taeniopygia* + *Melopsittacus*	AF1 Deletion: 2958	None	None
*Taeniopygia*	AF1 Deletion: 3318	AF3 Deletions: 578, 612-613	AF7 Deletions: 378, 407-417
*Melopsittacus*	AF1 Insertions: 231, 2239	AF3 Stop codon: 708-710	AF7 Insertion: 105
	AF1 Deletions: 432-433, 452-453, 504-505		AF7 Deletions: 91–94, 387
*Dasypus*	See Meredith et al. [2]	None	AF11 Deletion: 3’ end of intron 6, including the acceptor splice site, and the first 14 bp of exon 7 (665–678), which includes the last amino acid and the stop codon
*Choloepus*	See Meredith et al. [2]	Splice site mutation: AG to AT at intron 2 acceptor site (gb|ti|1338556193)	AF11 Deletions: 45–55, 600
			AF11 Initiation codon mutation: ATG to ACG (13–15)
			AF11 Stop codon: 356-358
*Orycteropus*	See Meredith et al. [2]	AF5 Deletions: 415-651 (all of exon 6), 1241-1253	AF11 Stop codons: 239–241, 299-301
		AF5 Stop codon: 388-390	

Numbers correspond to sequence alignment positions in Additional files. Additional file 1 = AF1; Additional file 3 = AF3; Additional file 5 = AF5; Additional file 7 = AF7; Additional file 9 = AF9; Additional file 11 = AF11. Cry = Cry SINE [47].

Blast searches with the virtual pseudogene mRNA of *Gallus ENAM* exon 9 recovered homologous fragments of this exon in *Meleagris gallopavo* (turkey), *Taeniopygia guttata* (zebra finch), *Anas platyrhynchos* (duck), and *Melopsittacus undulatus* (budgerigar). The 3’ region of *M. gallopavo ENAM* was recovered on chromosome 1, whereas the 5’ region was recovered on the Z chromosome of this species. All of the exon 9 sequences were characterized by frameshift mutations (Figure 1), including a 406 bp deletion near the 3’ end of the coding sequence of exon 9 that is shared by all five birds (Additional file 1).

*ENAM* sequences for *Orycteropus afer*, *Choloepus hoffmanni* and *Dasypus novemcinctus* were reported by Meredith et al. [2] and are not duplicated here. *Orycteropus ENAM* includes three frameshift mutations [2]. Among xenarthrans, *Choloepus ENAM* includes numerous frameshift mutations whereas *Dasypus ENAM* includes a single frameshift mutation that is located near the carboxy-terminal end of the coding sequence in exon 10 (= exon 9 of some non-mammalian vertebrates) [2].

### AMBN Gene

Blast searches with *Caiman crocodilus* (spectacled caiman) *AMBN* (AY043290) recovered portions of exons 1, 2, 3, 6, 8, 9, 10, and 11, in this order, on *Chrysemys* scaffold JH584398 (Additional files 3 and 4). The *AMBN* exons are located immediately upstream of *ENAM* exon 9 on scaffold JH584398. Frameshift mutations occur in exons 6, 10, and 11 (Figure 1, Table 2, Additional files 3, 4). *AMBN* sequences were not found in *Pelodiscus*.

Among birds, remnants of *AMBN* were discovered in *Anas* (scaffold 247: exons 2, 3, 5, 6, 7, 8), *Gallus* (Z chromosome: exons 2, 3, 5, 6)*, Taeniopygia* (Z chromosome; exons 6, 7, 8), and *Melopsittacus* (scaffold JH556633: exons 7, 8). Frameshift mutations occur in *Anas* (exons 2, 6, 8), *Gallus* (exon 3) and *Taeniopygia* (exon 6) (Additional file 3). A ten-bp frameshift deletion is shared by *Anas*, *Gallus*, and *Taeniopygia*, which together index the common ancestry of Neognathae, although this region was not recovered in *Meleagris* and *Melopsittacus*. There were no frameshifts in *Melopsittacus* exons 7 and 8, but a stop codon occurs in the latter exon.

Among mammals, we recovered exons 1–5, 7, and 10–13 in *Orycteropus afer* (Additional file 5). Exon 5 contains a stop codon and there is a 13-bp frameshift deletion in exon 13 (Table 2). Exon 6 has been deleted along with portions of introns 5 and 6 (Table 2). Exons 8 and 9 are 39-bp duplications of exon 7 that occur in primates [48] and their absence in *Orycteropus* is not unexpected. With the exception of *Elephantulus edwardii*, which has two duplications of exon 7 as in *Homo*, other afrotherians (*Loxodonta africana*, *Trichechus manatus*, *Procavia capensis*, *Chrysochloris asiatica*, *Echinops telfairi*) are similar to *Orycteropus* in possessing only exon 7. We also recovered sequences for exons 1–7 and 10–13 in *Dasypus AMBN* (Additional file 5). The recovered protein-coding sequence is intact, although the presumed start codon is ten codons downstream of the start codon in the human sequence and occurs in exon 2 rather than exon 1, as is also the case for *Loxodonta africana* (African elephant) (Additional file 6). Complete coding sequences for nine exons (1–5, 7, 10–12) and partial sequences for exons 6 and 13 were recovered from the *Choloepus* genome (Additional file 5). The putative start codon in *Choloepus* is located in exon 2 as in *Dasypus.* There are no frameshift mutations or stop codons in the available *Choloepus* sequence, but intron 2 exhibits an inactivating mutation at the acceptor splice site (AG to AT; see TI# 1338556193 in Trace Archives) (Table 2). By contrast, all of the donor and accepter splice sites exhibit canonical GT and AG motifs, respectively, in 16 mammals with enamel-capped teeth (taxon names provided in Methods).

Selection analyses based on an alignment of 20 mammalian *AMBN* sequences (Additional file 6) with codon frequency (CF) models 1, 2, and 3 all suggest elevated dN/dS ratios on the *Orycteropus* (CF1 = 0.71, CF2 = 0.77, CF3 = 0.89), *Choloepus* (CF1 = 0.86, CF2 = 0.90, CF3 = 1.04) and *Dasypus* (CF1 = 0.64, CF2 = 0.71, CF3 = 0.74) branches relative to the background rate (CF1 = 0.48, CF2 = 0.51, CF3 = 0.56) in mammalian taxa with enamel-covered teeth.

### AMEL Gene

Blast searches with *Paleosuchus palpebrosus* (Cuvier’s dwarf caiman) *AMEL* (AF095568) recovered most of exons 1, 3, 4, and 5 in *Pelodiscus*, with ancestral synteny preserved, on scaffold JH208023 (Additional files 7 and 8). Exons 1 and 5 were identified on scaffold JH584884 in *Chrysemys* (Additional files 7 and 8). *Chrysemys* and *Pelodiscus* both retain the same start codon as in crocodylians. Frameshift mutations are present in sequences of both testudine species (exon 1 in *Chrysemy*s, exons 1, 3, and 4 in *Pelodiscus*), although there are no shared frameshifts in these two exons (Figure 1, Table 2, Additional files 7 and 8). In addition, *Pelodiscus* and *Chrysemys* share exon-flanking sequences that are 3’ to exon 1 and 5’ to exon 5. The sequence that flanks exon 1 is > 1000 bp and includes regions that are homologous to intron 1 and possibly exon 2 of *Alligator mississippiensis* (Additional file 6). Intron 1 retains the canonical “GT” splice donor site in both testudines (Additional file 9). The homology of exon 2 is less certain, but nevertheless of potential importance as there are multiple shared frameshift mutations in *Chrysemys* and *Pelodiscus* (Additional file 9). The sequence that flanks exon 5 is > 600 bp long and is homologous to a portion of intron 4 of *A. mississippiensis* with the canonical “AG” splice acceptor site in *Chrysemys* (“AA” in *Pelodiscus*) (Additional file 10).

In birds, blast searches with *Gallus AMEL* pseudogene mRNA (EU340348) recovered homologous DNA sequences in *Anas* (exons 1–4 with original synteny on scaffold 415), *Meleagris* (exons 1–4 with original synteny on chromosome 1), *Melopsittacus* (exons 1–4 with original synteny on AGAI01063440), and *Taeniopygia* (exons 2–4 with original synteny on chromosome 1) (Additional file 7). All of the birds except for *Anas* share a common start codon with crocodylians. Frameshift mutations occur in all avian taxa, including a one-bp deletion in exon 4 that is shared by all five birds (Figure 1).

Among mammals, there are two stop codons in exon 6 of *Orycteropus AMEL* (Figure 1, Table 2, Additional file 11). *Choloepus AMEL* shows replacement of the methionine initiation codon in exon 2 by a threonine codon, an 11 bp frameshift deletion in exon 2, and a premature stop codon followed by a frameshift deletion in exon 6 (Figure 1, Table 2, Additional file 11). All of the inactivating mutations in *Choloepus* are corroborated by chromatograms in NCBI’s Trace Archives. The sequence for *Dasypus* is intact through exon 6 (excepting exon 4, see below), but a deletion incorporates the 3’ end of intron 6, including the acceptor splice site, and the first 14 bp of exon 7, including the stop codon. *Orycteropus*, *Choloepus*, and *Dasypus* also lack a functional copy of exon 4, either because of stop codons (*Orycteropus*, *Choloepus*) or a donor splice site mutation (GT to AT) in intron 4 that is adjacent to this exon. However, a functional copy of exon 4 is variably present in Mammalia [48] and is missing from six other afrotherians (*Chrysochloris, Echinops, Elephantulus, Loxodonta, Procavia, Trichechus*) with genome sequences because of stop codons, frameshift mutations, and/or splice site mutations (data not shown).

## Discussion

### EMP Genes in Turtles

Crown Testudines comprise 319 extant species [49], all of which are toothless and instead have a keratinized beak that in combination with strong jaw muscles allows testudines to tear food and capture prey [21]. The oldest testudine fossil is the Late Triassic *Odontochelys semitestacea* from China [25]. *Odontochelys* lacked a beak and instead retained both marginal and palatal teeth [25]. *Proganochelys quenstedti* is also known from the Late Triassic, and is more derived than *Odontochelys. Proganochelys* lacked marginal teeth, but palatal teeth were present. The morphology of the mandibles suggests that *Proganochelys* possessed a keratinized beak [26]. It remains unclear if palatal teeth, which occur on the vomer, palatine, and pterygoid bones, were lost in the common ancestor of all living turtles or independently in cryptodires and pleurodires owing to alternate phylogenetic hypotheses wherein taxa with palatal teeth such as *Kayentachelys apix* are positioned as stem testudines or as stem cryptodires [23,24,29-31,50,51]. In either case palatal teeth were lost no later than the Late Jurassic in the ancestry of crown testudines.

The retention of pseudogenized copies of three EMP genes (*AMEL*, *AMBN*, *ENAM*) in *Chrysemys* is perhaps surprising given the antiquity of tooth loss in turtles and the extended time window for large-scale deletions and/or rearrangements to erase or scramble the genomic instructions for enamel production in this edentulous taxon. However, remnants of *AMBN* and *ENAM* exon 9 in *Chrysemys* occur in juxtaposition to each other on the same contig. Kawasaki’s [52] reconstructions of SCPP gene order in both stem tetrapods and stem amniotes suggest that *AMBN* is immediately upstream of *ENAM*. Thus, it appears that remnants of this original gene order are present in the painted turtle genome. The protein-coding regions of *AMBN* and *ENAM* in *Chrysemys* have been battered by inactivating mutations, including SINE insertions in exon 9 of *ENAM*, but nevertheless retain unambiguous signatures of their heritage. The only EMP gene that was recovered in *Pelodiscus* is *AMEL*. It remains unclear whether *AMBN* and *ENAM* were completely deleted from the *Pelodiscus* genome during evolutionary history, are difficult to recognize because of numerous mutations, or are missing from the current assembly of the *Pelodiscus* genome due to incomplete sequencing assembly. *Pelodiscus AMEL* is relatively complete and includes exons 1, 3, 4, and 5 in their original syntenic order. By contrast, we only discovered exons 1 and 5 of *Chrysemys AMEL*. The two exons represent the only overlapping EMP fragments that were discovered in both *Pelodiscus* and *Chrysemys*. Frameshift mutations in *AMEL* occur in both species, but the only shared frameshift mutations occur in putative exon 2, which has equivocal homology with the same exon in crocodylians. Nevertheless, if these frameshift mutations are genuine then there is molecular evidence for the inactivation of enamel production in the common ancestry of Cryptodira.

The genetic and developmental basis of edentulism in turtles involves both tooth-specific and pleiotropic genes. Results presented here demonstrate that three tooth-specific EMP genes were pseudogenized in the ancestry of modern cryptodires. Previously, Tokita et al. [53] examined patterns of gene expression in the developing lower jaw of *Pelodiscus sinensis* and reported impairment of *Shh* signaling in the oral epithelium along with early-stage arrest of odontoblast development by abrogation of *Msx2* expression in dental mesenchyme. It remains unclear if changes in gene expression or EMP pseudogenization occurred first in the evolutionary history of turtles.

### EMP Genes in Birds

*AMEL* and *ENAM* pseudogenes have previously been reported in *Gallus*[20], which belongs to Galliformes. We provide extended evidence for inactivated EMP genes in birds and show that there are remnants of *AMEL* and *ENAM* in four other birds with complete genome sequences: *Meleagris* (Galliformes), *Anas* (Anseriformes), *Melopsittacus* (Psittaciformes), and *Taeniopygia* (Passeriformes) (Table 1). In addition, we recovered molecular evidence for an inactivated copy of *AMBN* in *Anas*, *Gallus*, *Taeniopygia*, and *Melopsittacus*. It is noteworthy that there are putative frameshift mutations in *AMBN*, *AMEL*, and *ENAM* that are shared by representatives of Galliformes (*Gallus*, *Meleagris*), Anseriformes (*Anas*), and Neoaves (*Taeniopygia*, *Melopsittacus*), which together index the deepest split in Neognathae [54]. It will be important to determine if these inactivating mutations are shared with Palaeognathae (tinamous and ratites), in which case molecular evidence would be consistent with the loss of enamel in the common ancestor of Neornithes rather than independently in Neognathae and Palaeognathae.

### EMP Genes in Mammals

It has previously been shown that *ENAM* is a pseudogene in a wide array of edentulous and enamelless mammals [1,2] (Table 1). Further, *AMEL* and *AMBN* sequences have been reported for baleen whales and in both cases are inactivated in multiple species [1,3]. However, the functional versus pseudogene status of *AMEL* and *AMBN* remains to be investigated in most edentulous and enamelless taxa.

Here, we provide evidence of inactivating mutations in *AMEL* in *Orycteropus afer* (aardvark) and *Choloepus hoffmanni* (Hoffmann’s two-toed sloth), both of which have enamelless teeth comprised of dentin. Multiple frameshift mutations in *Choloepus AMEL* are confirmed by Trace Archives chromatograms (Additional file 8). The two stop codons in *Orycteropus* are based on Illumini Hi-Seq sequencing technology with 44X genome coverage.

Previous studies suggest that *AMBN* may play a role in dentin formation/regeneration [55,56]. However, this hypothesis is contradicted by our finding that there are inactivating mutations in *Orycteropus* and *Choloepus AMBN*. Moreover, dN/dS ratios indicate relaxed purifying selection in these taxa. Similarly, McGowen [19] reported a premature stop codon in exon 13 (JF504758) of *Monodon monoceros* (narwhal). Narwhals have vestigial teeth, and in the case of males a single enlarged tusk. Both the vestigial teeth and tusk are composed of dentin and cementum, but no enamel [57-59]. The occurrence of a premature stop codon in *Monodon AMBN*, in conjunction with inactivating mutations in two other lineages with enamelless teeth (*Orycteropus*, *Choloepus*), is consistent with the hypothesis that the only essential, non-redundant role of *AMBN* is in enamel formation. Along these lines, ameloblastin knockout mice do not form an enamel layer, and lack dentin defects [13,15].

By contrast with *Choloepus*, where enamel loss occurred more than 50 million years ago, enamel degeneration in *Dasypus* occurred more recently [2]. *Dasypus* retains a vestigial enamel or enamel-like substance that covers the dentin of some teeth and is quickly worn off. Moreover, there are no frameshifts in *ENAM* that are shared by *Dasypus* and other armadillo genera [2]. The single frameshift in *Dasypus ENAM* precludes a functional, full-length enamelin protein, but the location of the frameshift is close to the 3’ end of the protein-coding sequence in exon 10, and shorter protein products are possible [2]. Our finding that *AMBN* is intact in *Dasypus*, and that only the last amino acid is truncated from the coding region of *AMEL*, is consistent with the presence of vestigial ‘enamel’ in this species [2]. We also note that a partial *AMBN* sequence (JF701624) for *Kogia breviceps* (pygmy sperm whale) has an intact protein-coding region even though *K. breviceps* has enamelless teeth and frameshift mutations in the *ENAM* gene. It will be important in future studies to sequence the remaining exons of *Kogia AMBN* to determine if the missing exonic regions contain frameshift mutations and/or other inactivating mutations.

## Conclusions

Edentulism has evolved independently in multiple lineages of living amniotes including turtles, birds, echidnas, baleen whales, anteaters, and pangolins. There are also mammals with enamelless teeth including pygmy sperm whale, narwhal, sloths, armadillos, and aardvarks. In every case these edentulous or enamelless forms have descended from ancestors with enamel-capped teeth. Thus, amniote diversity provides a natural laboratory for testing hypotheses of tooth-specific gene function [3]. Moreover, this laboratory includes multiple, replicated experiments. *AMBN*, *AMEL*, and *ENAM* have all been postulated to have tooth-specific or even enamel-specific gene functions [11-17], although pleiotropic functions have been suggested for *AMBN*[9,48,60,61] and *AMEL*[62,63]. The widespread occurrence of EMP pseudogenes in turtles, birds, and several mammalian lineages (Table 1) provides compelling evidence that the only unique, non-redundant function of these genes in amniotes is in enamel formation: functional copies of these genes have not been retained by natural selection when enamel production was abrogated independently in distantly related lineages. By contrast, representative sequences from amniotes with enamel-capped teeth retain intact coding sequences for *AMBN*[1,48]; also see Additional file 11, *AMEL*[3,48,64], and *ENAM*[1,2,18,37,65].

The evolution of tooth loss in multiple amniote lineages also provides a model system for integrating the fossil record, phylogenetics, and genomics. This system allows for reciprocal hypothesis testing and provides a multifaceted, synthetic view on macroevolutionary transitions in testudines, birds, and edentulous/enamelless mammals [1,2]. The fossil record and phylogenetics combine to predict the occurrence of molecular fossils of tooth-specific genes in the genomes of edentulous and enamelless amniotes, and molecular fossils have been discovered in all lineages that have been investigated. We suggest that these striking patterns may prove especially useful for educating the public on the convergence of evidence that clearly documents the evolutionary process over deep time.

## Competing interests

The authors declare that they have no competing financial interests.

## Authors’ contributions

JG, RWM, and MSS conceived and designed the study. RWM and MSS performed the analyses. MSS wrote the manuscript. JG and RWM provided comments on the manuscript. JG and MSS prepared figures. All authors read and approved the final manuscript.

## Supplementary Material

Additional file 1 **Nexus alignment of *ENAM *exon 9 sequences for two crocodylians, one testudine, and five birds. **The stop codon in crocodylians and *Chrysemys picta *occurs at positions 3788–3790. A 406 bp deletion that is shared by all five birds occurs near the 3’ end of the coding region at positions 3353–3758. Additional frameshift mutations are shared by four of five birds including representatives of Anseriformes, Galliformes, and Passeriformes (2634–2637). Cry SINE elements [47] in *Chrysemys *occur at positions 994–1138 and 4782–4994. Several long strings of Ns in *Chrysemys picta *were abridged to shorter strings of ten Ns. Click here for file

Additional file 2 **Schematic alignment showing regions of *ENAM *exon 9 that were identified in *Chrysemys picta*. **Green bars below sequence numbers show regions of sequence similarity between *C. picta *and *Alligator mississippiensis*. Red rectangles = frameshift mutations in *C. picta*; light blue rectangles = indels within the coding sequence that are in multiples of three base pairs; CRY = Cry SINE insertion; red arrow = position of stop codon in *A. mississippiensis*. Click here for file

Additional file 3 **Nexus alignment of *AMBN *exon sequences for two crocodylians, one testudine, and one bird. **The start and stop codons in crocodylians occur at positions 127–129 (exon 2) and 1374–1376 (exon 11), respectively. Click here for file

Additional file 4 **Schematic alignment showing regions of *AMBN *exons 1–11 that were identified in *Chrysemys picta*.** Green bars below sequence numbers show regions of sequence similarity between *C. picta *and *Alligator mississippiensis*. Dark blue rectangles = numbered exons in *A. mississippiensis*; red rectangles = frameshift mutations in *C. picta*; light blue rectangles = indels within the coding sequence that are in multiples of three bp; dark blue arrow = position of start codon in *A. mississippiensis*; red arrow = position of stop codon in *A. mississippiensis*. Click here for file

Additional file 5 **Nexus alignment of *AMBN *exon sequences for*Homo sapiens*, *Orycteropus afer*and six other afrotherians, *Dasypus novemcinctus*, and *Choloepus hoffmanni*. **The start and stop codons in *Homo* occur at positions 121–123 (exon 1) and 1468–1470 (exon 13), respectively. The presumed start codon in *Dasypus* occurs at positions 151–153. *Dasypus* shares the same stop codon as *Homo *at positions 1468–1470, whereas the presumed stop codon in *Choloepus *is at positions 1477–1479. Click here for file

Additional file 6 **Nexus alignment of protein-coding *AMBN *sequences for 20 mammals that was used in selection analyses with PAML 4.5 **[36]**.** Click here for file

Additional file 7 **Nexus alignment of *AMEL *exon sequences in nexus format for two crocodylians, two testudines, and five birds. ** The start and stop codons in crocodylians occur at positions 69–71 (exon 1) and 731–733 (exon 5), respectively. A putative one-bp frameshift deletion that is shared by all five birds species occurs at position 242 in exon 4. Click here for file

Additional file 8 **Schematic alignment showing regions of *AMEL *exons 1–5 that were identified in *Chrysemys picta *and *Pelodiscus sinensis *. **Bars below sequence numbers show regions of sequence similarity between *Alligator mississippiensis *and both testudines (green) or *A. mississippiensis *and one testudine (chartreuse). Dark blue rectangles = numbered exons in *A. mississippiensis*; red rectangles = frameshift mutations in *C. picta*; light blue rectangles = indels within the coding sequence that are in multiples of three bp; dark blue arrow = position of start codon in *A. mississippiensis*; red arrow = position of stop codon in *A. mississippiensis*. Click here for file

Additional file 9 **Nexus alignment of *Alligator mississippiensis AMEL *exon 1 (positions 1–114), intron 1 (positions 115–813), and exon 2 (838–894) with exon 1 and flanking sequences from *Chrysemys picta *and *Pelodiscus sinensis*.** Click here for file

Additional file 10 **Nexus alignment of *AMEL *intron 4 (positions 1–662) and exon 5 (positions 663–844) sequences for *Chrysemys picta *and *Pelodiscus sinensis*.** Click here for file

Additional file 11 **Nexus alignment of *AMEL *sequences (exons 2–7) for *Homo sapiens*, *Orycteropus afer *and six other afrotherians (*Echinops telfairi, Chrysochloris asiatica, Elephantulus edwardii, Trichechus manatus, Loxodonta africana, Procavia capensis*), *Dasypus novemcinctus*, and *Choloepus hoffmanni*. **The start and stop codons in *Homo *occur at positions 13–15 (exon 2) and 668–670 (exon 7), respectively. The mutated stop codon and frameshift mutation in exon 2 of *Choloepus *are clear in gnl|ti|1325289135 and gnl|ti|1311883086. The stop codon in exon 4 of *Choloepus *is confirmed by traces gnl|ti|1325336699 and gnl|ti|1312095734. The frameshift mutation in exon 6 of *Choloepus *is evident in trace gnl|ti|1363579107. The deletion that includes the 3’ region of intron 6 and first 14 bp of exon 7 in *Dasypus *is evident in gn|ti| 1886397515. Click here for file
