# Supplementary material for: Molecular decay of enamel matrix protein genes in turtles and other edentulous amniotes
Source: BMC Evol Biol. 2013 Jan 23;13:20. doi: 10.1186/1471-2148-13-20 (PMC3562159; doi:10.1186/1471-2148-13-20)
Supplement: Additional file 2 — Schematic alignment showing regions of ENAM exon 9 that were identified in Chrysemys picta. Green bars below sequence numbers show regions of sequence similarity between C. picta and Alligator mississippiensis. Red rectangles = frameshift mutations in C. picta; light blue rectangles = indels within the coding sequence that are in multiples of three base pairs; CRY = Cry SINE insertion; red arrow = position of stop codon in A. mississippiensis. [file 1471-2148-13-20-S2.pdf]

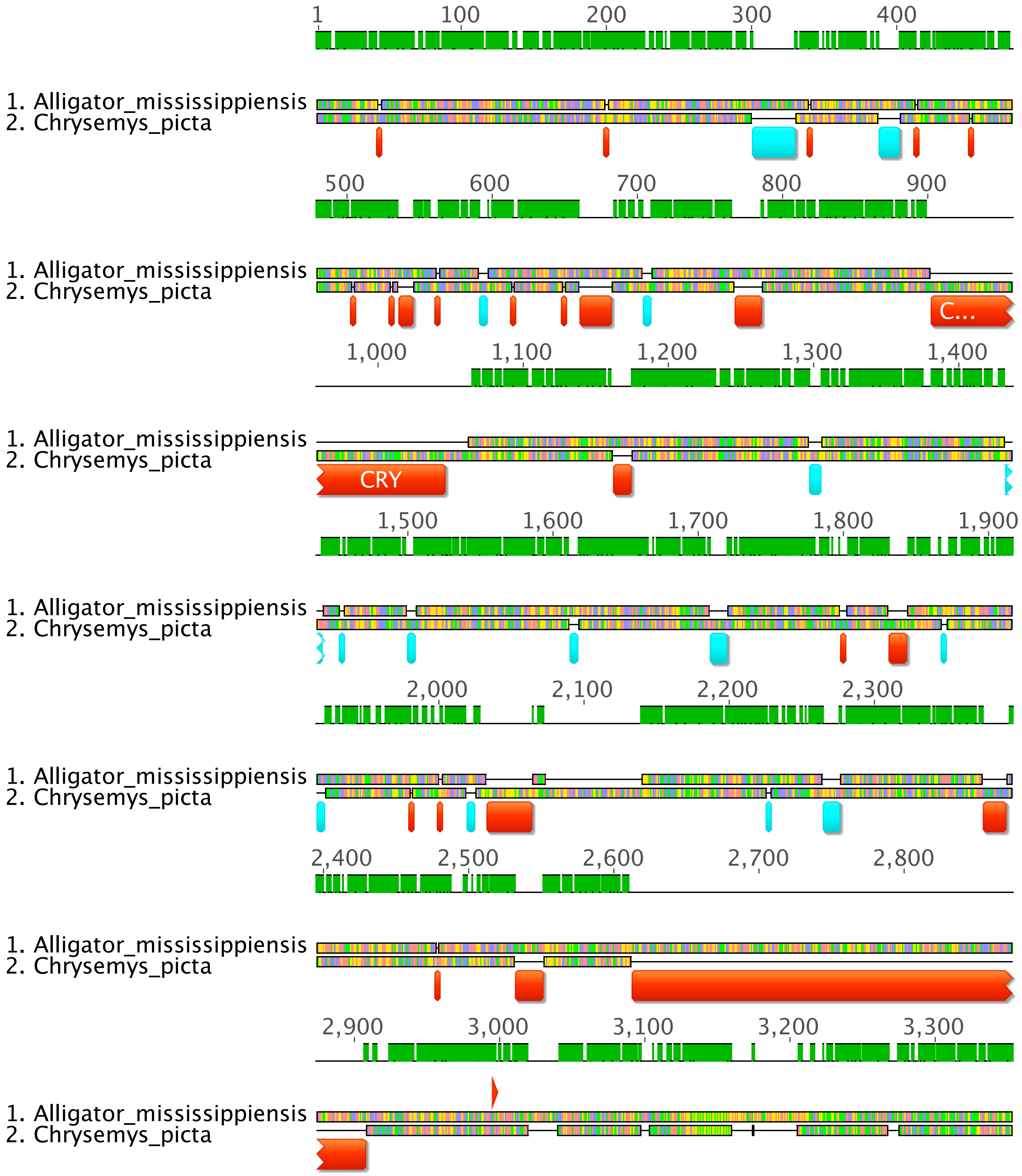
1
100
200
300
400
1. Alligator_mississippiensis
2. Chrysemys_picta
500
600
700
800
900
1. Alligator_mississippiensis
2. Chrysemys_picta
C...
1,000
1,100
1,200
1,300
1,400
1. Alligator_mississippiensis
2. Chrysemys_picta
CRY
1,500
1,600
1,700
1,800
1,900
1. Alligator_mississippiensis
2. Chrysemys_picta
2,000
2,100
2,200
2,300
1. Alligator_mississippiensis
2. Chrysemys_picta
2,400
2,500
2,600
2,700
2,800
1. Alligator_mississippiensis
2. Chrysemys_picta
2,900
3,000
3,100
3,200
3,300
1. Alligator_mississippiensis
2. Chrysemys_picta

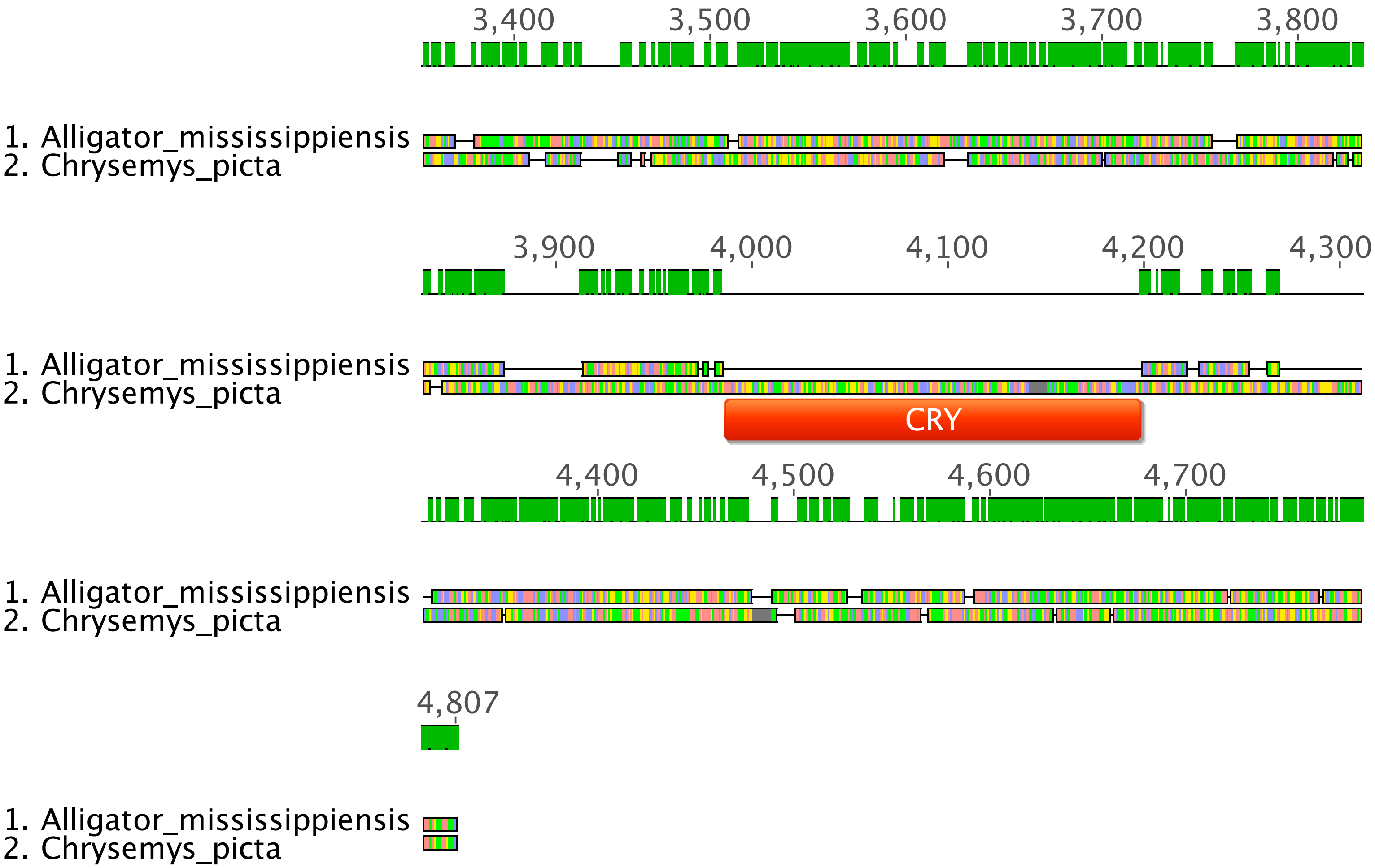

3,400
3,500
3,600
3,700
3,800
1. Alligator_mississippiensis
2. Chrysemys_picta
3,900
4,000
4,100
4,200
4,300
1. Alligator_mississippiensis
2. Chrysemys_picta
CRY
4,400
4,500
4,600
4,700
1. Alligator_mississippiensis
2. Chrysemys_picta
4,807
1. Alligator_mississippiensis
2. Chrysemys_picta
