# Supplementary figures and images for: Molecular decay of enamel matrix protein genes in turtles and other edentulous amniotes
Source: BMC Evol Biol. 2013 Jan 23;13:20. doi: 10.1186/1471-2148-13-20 (PMC3562159; doi:10.1186/1471-2148-13-20)

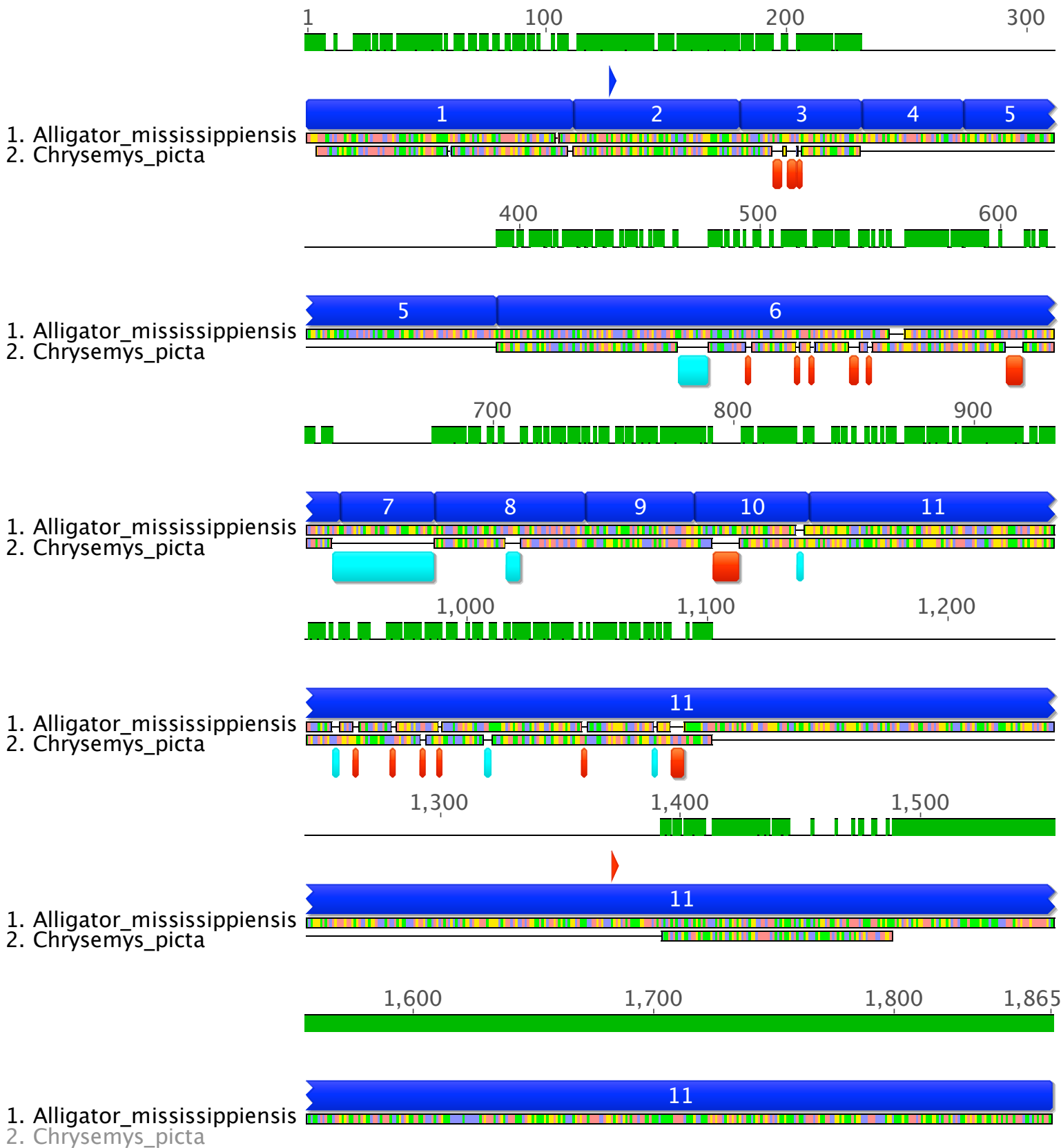

Supplement: Additional file 4 — Schematic alignment showing regions of AMBN exons 1–11 that were identified in Chrysemys picta. Green bars below sequence numbers show regions of sequence similarity between C. picta and Alligator mississippiensis. Dark blue rectangles = numbered exons in A. mississippiensis; red rectangles = frameshift mutations in C. picta; light blue rectangles = indels within the coding sequence that are in multiples of three bp; dark blue arrow = position of start codon in A. mississippiensis; red arrow = position of stop codon in A. mississippiensis. [file 1471-2148-13-20-S4.pdf]

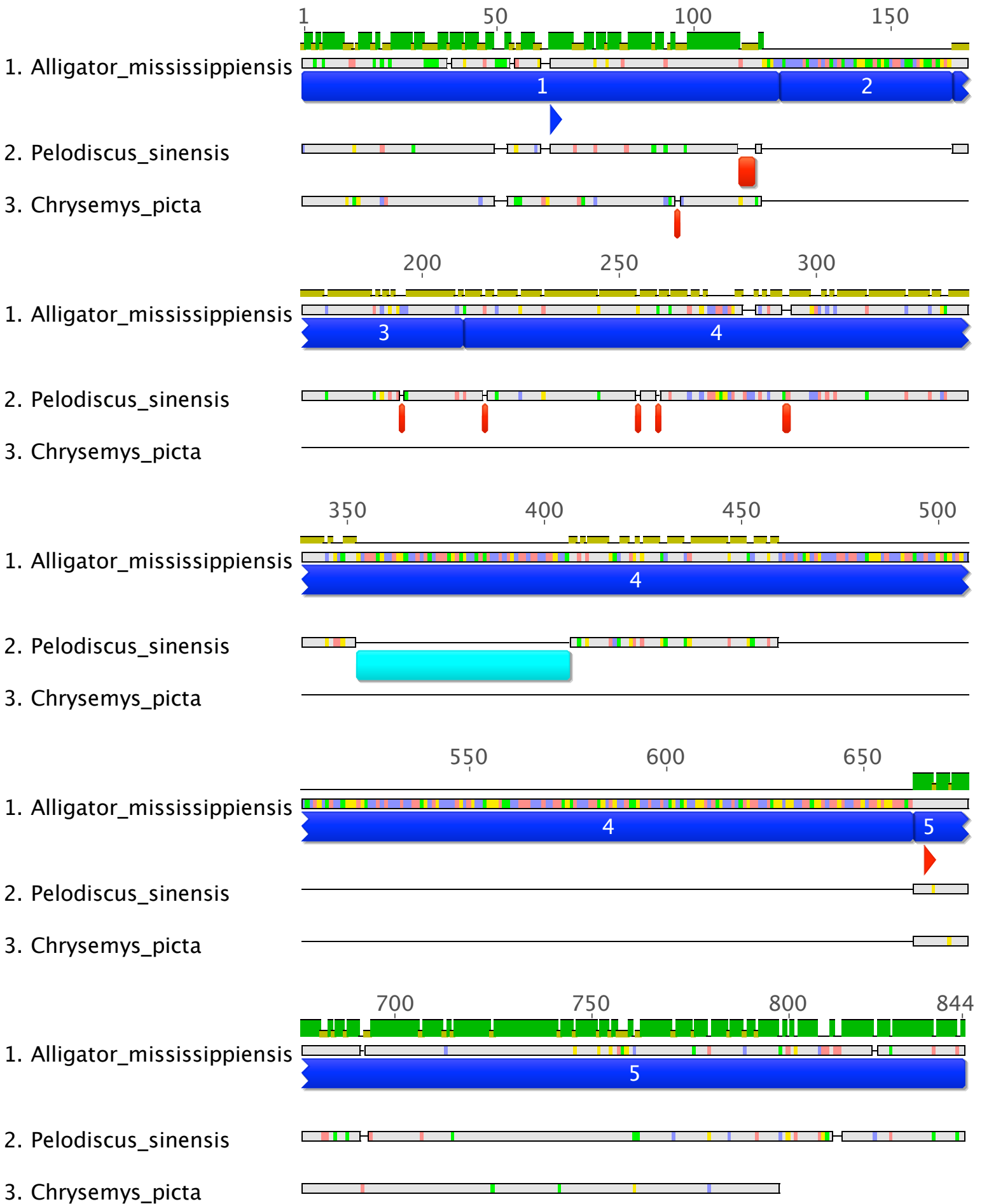

Supplement: Additional file 8 — Schematic alignment showing regions of AMEL exons 1–5 that were identified in Chrysemys picta and Pelodiscus sinensis . Bars below sequence numbers show regions of sequence similarity between Alligator mississippiensis and both testudines (green) or A. mississippiensis and one testudine (chartreuse). Dark blue rectangles = numbered exons in A. mississippiensis; red rectangles = frameshift mutations in C. picta; light blue rectangles = indels within the coding sequence that are in multiples of three bp; dark blue arrow = position of start codon in A. mississippiensis; red arrow = position of stop codon in A. mississippiensis. [file 1471-2148-13-20-S8.pdf]
